# Epigenetic functions enriched in transcription factors binding to mouse recombination hotspots

**DOI:** 10.1186/1477-5956-10-S1-S11

**Published:** 2012-06-21

**Authors:** Min Wu, Chee-Keong Kwoh, Teresa M Przytycka, Jing Li, Jie Zheng

**Affiliations:** 1 School of Computer Engineering, Nanyang Technological University, Singapore; 2 Computational Biology Branch, NCBI, NLM, National Institutes of Health, USA; 3 EECS Department, Case Western Reserve University, USA

## Abstract

The regulatory mechanism of recombination is a fundamental problem in genomics, with wide applications in genome-wide association studies, birth-defect diseases, molecular evolution, cancer research, etc. In mammalian genomes, recombination events cluster into short genomic regions called "recombination hotspots". Recently, a 13-mer motif enriched in hotspots is identified as a candidate cis-regulatory element of human recombination hotspots; moreover, a zinc finger protein, PRDM9, binds to this motif and is associated with variation of recombination phenotype in human and mouse genomes, thus is a trans-acting regulator of recombination hotspots. However, this pair of cis and trans-regulators covers only a fraction of hotspots, thus other regulators of recombination hotspots remain to be discovered. In this paper, we propose an approach to predicting additional trans-regulators from DNA-binding proteins by comparing their enrichment of binding sites in hotspots. Applying this approach on newly mapped mouse hotspots genome-wide, we confirmed that PRDM9 is a major trans-regulator of hotspots. In addition, a list of top candidate trans-regulators of mouse hotspots is reported. Using GO analysis we observed that the top genes are enriched with function of histone modification, highlighting the epigenetic regulatory mechanisms of recombination hotspots.

## Introduction

Recombination is one of the most fundamental processes in molecular biology, and is under intense research in genomics. In many species, recombination events are clustered into narrow genomic regions (usually a few kb long) called "recombination hotspots". During meiosis, recombination events are required to ensure correct segregation of homologous chromosomes, and thus abnormality or absence of meiotic recombination can lead to aneuploidy disorders such as Down syndrome. In addition to mutations, recombination is an important evolutionary force that shapes the linkage disequilibrium (LD) patterns in human genetic variation; as a result, hotspots tend to overlap with boundaries of haplotype blocks, which is a key observation underlying genome-wide association studies (GWAS) and the HapMap project [1]. Therefore, an increased understanding of the mechanism of recombination hotspots would shed light on various important aspects in molecular biology and medicine, such as genome instability, disease gene mapping, molecular evolution, etc. Despite the importance of recombination hotspots, many questions remain open, such as the regulatory mechanisms of the locations and activities of hotspots.

Recently, breakthroughs have been made to discover the regulatory mechanisms of meiotic recombination hotspots in mammalian gnomes. In 2010, three Science papers [2-4] reported the identification of PRDM9 gene as a trans-regulator of recombination hotspots in human and mouse genomes. PRDM9 is a zinc finger protein that binds to DNA, and its binding site contains a 13-mer motif previously found to be enriched in human hotspots [5]. Using an LD-based approach named LDsplit, Zheng et al. [6] identified HapMap SNPs (single nucleotide polymorphisms) in human chromosome 6 that are associated with recombination hotspots, and confirmed the sperm typing experimental result on DNA2 hotspot [7]. Importantly, proximal to the SNPs identified by LDsplit, Zheng et al. found an enriched 11-mer motif which partially matches the aforementioned 13-mer motif in the binding site of PRDM9 [6]. Using Chip-Seq data, Smagulova et al. [8] analyzed the molecular features of mouse recombination hotspots, and observed that a consensus motif enriched in mouse hotspots aligns with the predicted binding site of mouse PRDM9 significantly. These exciting discoveries are promising to integrate previously separate observations into one picture.

It has been observed that, despite over 99% sequence identity between the human and chimpanzee genomes, the positions of recombination hotspots are rarely conserved between the two species [9]. This puzzle has been partially answered by Myers et al. [2], who found that, as PRDM9 evolves rapidly, its binding sites are very different between human and chimpanzee. The "hotspot paradox" states that due to biased gene conversion a hotspot tends to kill itself, nevertheless, there remain many hotspots in extant genomes [10]. This paradox may be explained by the rapid evolution of PRDM9 as well, i.e. many new hotspots can be generated in a short time by a few mutations in the zinc finger binding array of PRDM9. It is believed that epigenetic mechanisms play key roles in the regulation of meiotic recombination. PRDM9 is a transcription factor with epigenetic functions (e.g. histone H3K4 trimethyltransferase activity). Importantly, PRDM9 is uniquely expressed in early meiosis and its deficiency is associated with sterility, which coincides with the association of meiotic recombination hotspots with birth-defect diseases. However, it is estimated that PRDM9 can explain only 18% of variations in human recombination phenotype [3], and the 13-mer motif covers only 41% of human hotspots [5]. Therefore, PRDM9 is unlikely to be the only trans-regulator of recombination hotspots. To carry out recombination accurately, it must function in concert with other proteins to form a regulatory pathway. Hence, it is highly motivated to discover other genes and regulatory pathways regulating recombination hotspots.

The approaches to the discovery of PRDM9 and recent related works on recombination hotspots [11] typically search for motifs enriched in hotspots, and then search for proteins that may bind to the motifs. Although successful in the discovery of PRDM9, this approach has a few limitations. First, unsupervised motif-finding is a notoriously difficult problem, and motifs found in this way tend to be short due to the limited power of motif-finding algorithms and large amounts of sequence data. Second, it may be difficult to infer the protein that binds to a short enriched motif, either because the enrichment of the motif is not due to the binding of a trans-regulator of hotspots, or because multiple proteins bind to the same motif. Last but not least, the procedure of identifying PRDM9 is a manual process that requires biochemical and genetic knowledge rather than an automatic discovery in large scale. The emergence of high-throughput genomic data of more human populations and other species calls for an efficient automatic procedure for discovering trans-regulators like PRDM9. It is our goal to develop such a method of genome-wide discovery of trans-regulators of recombination hotspots.

**Figure 1:**
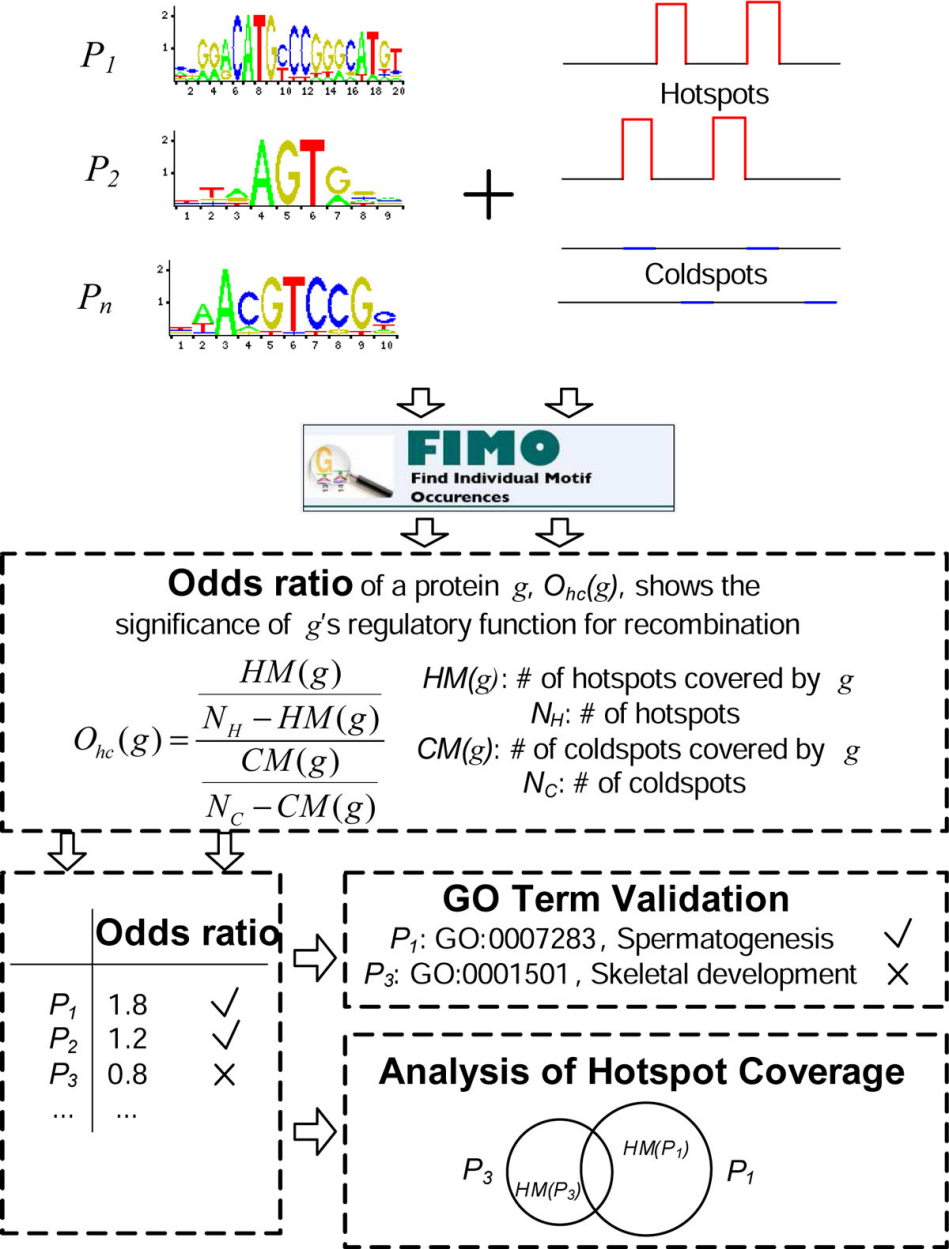
**The flowchart of our method for predicting tran-regulators of recombination hotspots**. Figure 1 shows the framework for predicting tran-regulators of recombination hotspots.

In this paper, we propose an approach to discovering trans-regulatory proteins similar to PRDM9 of recombination hotspots in mouse genome. Instead of starting from short sequence motifs enriched in hotspots, we scan the binding sites of DNA-binding proteins (e.g. transcription factors) across DNA sequences of hotspots and coldspots. As shown in Figure 1, the statistical score based on the enrichment of target binding sites is designed to predict the likelihood of each protein to initiate recombination. Moreover, a novel method is designed to identify the Gene Ontology (GO) terms that are shared by candidate trans-regulators. Applying this pipeline of knowledge discovery on a genome-wide map of mouse hotspots recently published [8], we first confirmed that PRDM9 is a major trans-regulator of mouse hotspots. Second, we identified a list of top candidate trans-regulators of mouse hotspots. Interestingly, our GO analysis shows that the candidate regulators predicted as such are enriched with the function of histone modification, high-lighting the epigenetic regulatory mechanisms known to be key in recombination hotspots. Our method can be used for the automatic discovery of trans-regulators in addition to PRDM9 on new genetic data and other species. The results in this paper not only confirm the discovery of PRDM9 gene, but also provide new candidate proteins to guide further experimental studies of recombination hotspots.

## Methods

In this section, we introduce our method for predicting tran-regulators as shown in Figure 1. Firstly, we collect mouse TFs and their binding motifs. Secondly, we define odds ratio for these TFs showing their preference to bind to hotspots by analyzing FIMO search results. This odds ratio is then utilized to show the significance of each TF's regulatory function for recombination. Lastly, we perform GO term validation and analysis of hotspot coverage for our candidate trans-regulators.

### Deriving consensus motifs

Persikov et al. [12] proposed a method to predict the binding between a DNA motif and a given protein using support vector machines (SVM). Myers et al. [2] first applied this method to generate some approximate motifs as candidates. Each candidate motif will be assigned a score showing its propensity to bind with the given protein. Since the potential interactions between zinc fingers were not taken into consideration in [12], Myers et al. then continued to maximize the score of a candidate binding motif by successively changing single bases within it. Final binding motifs were then obtained when no score increases for them. The predicted mouse PRDM9 binding sequence and degeneracy are shown in Figure S5 in the supporting online material of [2]. We thus take this predicted PRDM9 binding sequence as our first consensus motif.

Transcription factor (TF) binding affinities are typically modeled as position frequency matrices and JASPAR database (http://jaspar.genereg.net) [13] provides open-access for matrix profiles describing the DNA-binding patterns of TFs. The current release of JASPAR database holds 457 non-redundant, curated matrix profiles. For example, there are 53 for mouse, 75 for human and 117 for yeast. These 53 matrix profiles for mouse TFs are used in this paper to predict trans-regulators of mouse hotspots. In addition, we also extracted the matrix profiles for 118 TFs from TRANSFAC database [14] for our experiments and analysis.

### Protein binding preference in hotspots

For the above binding motifs, we employ the software tool FIMO [15] to scan for their occurrences in both hotspots and coldspots. FIMO takes two files as inputs, namely, a file containing one or more query motifs and another file as the sequence database. Particularly, each query motif is represented as a position-specific frequency matrix and the sequence database consists of known hotspots and our generated coldspots (see the Results section for more details about coldspot generation). FIMO computes a log-likelihood ratio score for each position of the given sequence database and converts this score to p-value and q-value to show the statistical significance of this position. Finally, FIMO outputs a ranked list of motif occurrences, each of them associated with a log-likelihood ratio score, p-value and q-value.

Using the numbers of motif occurrences in hotspots and coldspots, as output by FIMO search, we measure the preference of a protein to bind in hotspots with the odds ratio *O_hc _*= (*HM/HN*) */*(*CM/CN*). Here, *HM *is the number of hotspots with at least one motif occurrence (i.e. a hit of FIMO search), *HN *is number of hotspots without any hit (i.e. *HN *= *N_H _*- *HM*, *N_H _*is the number of hotspots as shown in Figure 1), *CM *is the number of coldspots with at least one hit, and *CN *is the number of coldspots without any hit (i.e. *CN *= *N_C _*- *CM*, *N_C _*is the number of coldspots). This odds ratio measures the relative risk associated with the presence of a binding motif in hotspots compared to coldspots. Hereafter, we will use the odds ratio *O_hc _*to measure the likelihood that a protein is a trans-regulator of recombination hotspots.

### Finding associated GO terms

Given a gene *g*, *T *(*g*) is the set of GO terms annotating this gene. We define the similarity between a term *t *and a gene *g*, *S*(*t*, *g*), in equation 1 and subsequently define the similarity between *t *and a set of genes *G*, *S*(*t*, *G*), in equation 2.

(1) $$S\left(t,g\right)\;=\frac{1}{\left|T\left(g\right)\right|}\underset{t^{\prime }\in T\left(g\right)}{\sum }sim\left(t,t^{\prime }\right)$$

(2) $$S\left(t,G\right)\;=\frac{1}{\left|G\right|}\underset{g\in G}{\sum }S\left(t,g\right)$$

Here, *sim*(*t*, *t*') in equation 1 is the semantic similarity between GO terms *t *and *t*' and we applied the method in [16] to calculate *sim*(*t*, *t'*).

Let *HG *denote the sets of genes with high odds ratio scores (candidate trans-regulators) and *G *be the whole set of genes we considered. The scores *S*(*t*, *HG*) and *S*(*t*, *G*) can be finally utilized to show *t*'s enrichment in *HG*. More specifically, their gap with respect to the term *t*, *gap*(*t*) in equation 3, can be used to discriminate *t*'s enrichment in *HG*. For example, a large gap indicates that *t *is enriched in the genes with high odds ratio while not enriched in the whole set of genes.

(3) $$gap\left(t\right)=\frac{S\left(t,HG\right)-S\left(t,G\right)}{S\left(t,G\right)}$$

## Results

### Experimental data

**Figure 2:**
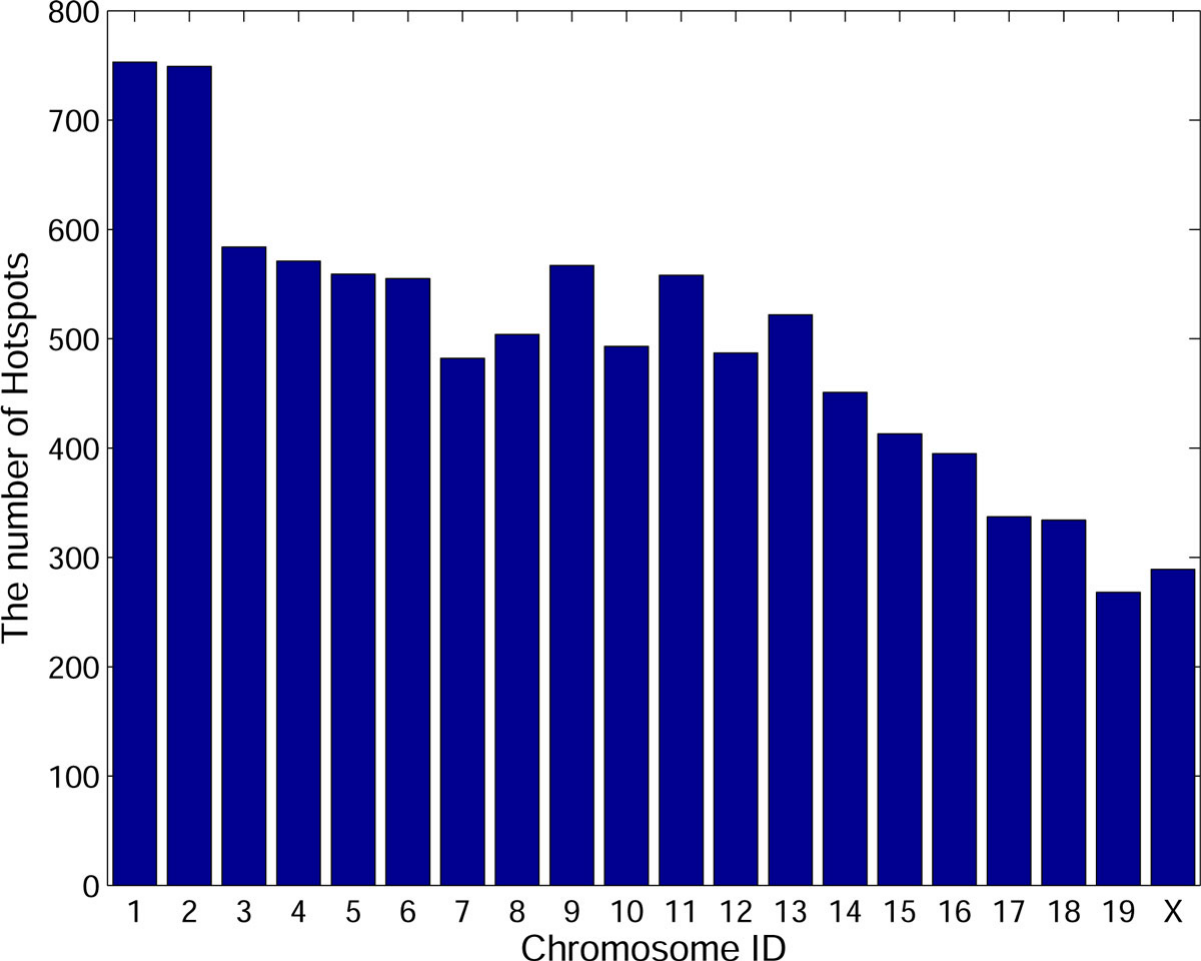
**The distribution of hotspots in each chromosome**. Figure 2 shows the number of hotspots for each chromosome.

We downloaded the mouse recombination hotspots from [8]. There are 9874 hotspots in all and the average hotspot width is 3414.08b. Figure 2 shows the distribution of hotspots in each chromosome. For example, chromosome 1 has the largest number of hotspots (753 hotspots). According to the hotspot boundaries, we extracted their DNA sequences from mouse genome and mouse DNA sequences (version: MGSCv37) were downloaded from NCBI.

Then, as statistical control we selected coldspots that have the following properties. First, coldspots have the same size and distribution as hotspots. Second, each coldspot is at least 50kb far away from other hotspots. Third, any two coldspots do not have common sequences--they are non-overlapping.

In addition, the GO data for GO term analysis were downloaded from [17].

### Enrichment of PRDM9 binding in mouse genome

First, we applied our method on the PRDM9 protein, which has been recently discovered as a trans-acting regulator of meiotic recombination hotspots, and is under intense research. The binding sequence of mouse PRDM9 was a 33-mer obtained from [2]. A matrix representing the degeneracy of mouse PRDM9 binding motif was fed into FIMO to search in the DNA sequences of hotspots and coldspots. To get more reliable estimation on coldspots, we randomly selected and searched by FIMO on coldspots for 20 times and then counted the average numbers of motif occurrences over the 20 runs.

**Figure 3:**
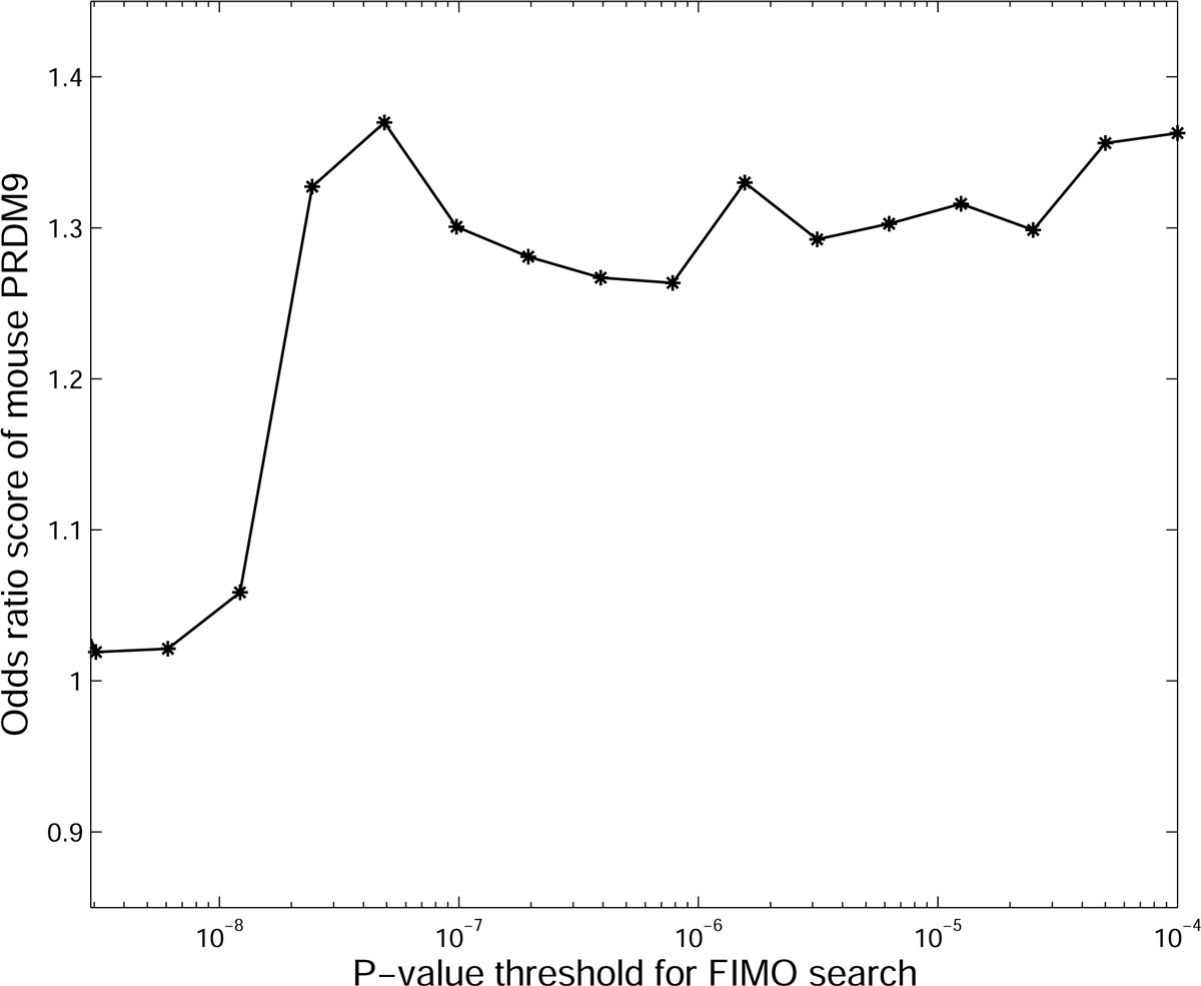
**The odds ratio scores of mouse PRDM9 with different p-value thresholds for FIMO search**. When we used different p-value thresholds for FIMO search, we will get different odds ratio scores. Figure 3 shows the impact of the p-value threshold on the odds ratio scores of mouse PRDM9.

Besides the query motif and the sequence database, FIMO will have an additional input, i.e., the p-value threshold for the output motif occurrences (a motif occurrence here refers to the binding between the query motif and background sequence). All the motif occurrences with p-values lower than the threshold will be considered to be reliable. Figure 3 shows the odds ratio scores of PRDM9 using different p-value thresholds for FIMO search. We can find that the odds ratio scores of PRDM9 are quite stable (around 1.3) when the threshold is in the range [3.0 × 10^-7^, 1.0 × 10^-4^]. This indicates that a medium setting of the p-value threshold will provide us a stable and reliable estimation of odds ratio score. Finally, we set the p-value threshold as 3.73×10^-6 ^which is in the above range (We also use this threshold for FIMO search on the following JASPAR database and TRANSFAC database) so that all the output motif occurrences are statistically meaningful with q-values less than 0.05. In this case, the numbers of occurrences of mouse PRDM9 motif are shown in Table 1. It is obvious that the binding sites of PRDM9 are more enriched in hotspots than coldspots, as demonstrated by the odds ratio *O*_*hc *_= 1.30 with p-value less than 10^-4 ^using *χ*^2 ^test with Yates' correction. These results are supportive to the discovery of PRDM9 as a trans-regulator for recombination hotspots [2-4].

**Table 1:** Number of occurrences of mouse PRDM9 motif (FIMO, q-value *<*0.05) on hotspots and coldspots

	# hits	# regions with hit(s)	# regions without hit
Hotspots	4954	1405	8469
Coldspots	4598.8	1120.35	8753.65

### Other TFs with binding sites enriched in hotspots

Encouraged by the positive results on PRDM9 obtained using our approach, we analyzed other mouse transcription factors (TFs) from JASPAR database [13], in hope of identifying proteins with enriched binding sites in hotspots vs. coldspots. From JASPAR database, we downloaded the degeneracy matrices of 53 TFs, which are input to FIMO to search hits in mouse hotspots and coldspots.

Table 2 shows the numbers of FIMO hits for all the 12 mouse TFs whose odds ratio scores *O_hc _*are larger than 1.20. The last column shows the p-values of the odds ratios using *χ*^2 ^test with Yates' correction. All the odds ratios in this table have p-values less than 0.05, indicating that they all are statistically significant. In addition, out of all 21 TFs with odds ratios larger than 1, 17 TFs have statistically significant odds ratios with p-values less than 0.05. All these 12 genes in this table will be considered as candidate trans-regulators and utilized for the further GO term analysis.

**Table 2:** The numbers of FIMO hits of mouse TFs from JASPAR database with top 12 *O*_*hc*_, ranked by the p-values of the odds ratios (PRDM9's results are also shown in this table)

*HG *Genes	*HM*	*HN*	*CM*	*CN*	*O_hc_*	p-value
KLF4	886	8988	643.15	9230.85	1.415	*<*0.0001
ZFX	437	9437	329	9545	1.343	*<*0.0001
CTCF	1002	8872	769.9	9104.1	1.336	*<*0.0001
PRDM9	1405	8469	1120.35	8753.65	1.30	*<*0.0001
RXRA	1015	8859	819.55	9054.45	1.266	*<*0.0001
ESRRB	792	9082	663.4	9210.6	1.211	0.0002
GABPA	110	9764	67.05	9806.95	1.648	0.0006
MYCN	197	9677	137.45	9736.55	1.440	0.0006
SPZ1	322	9552	249.55	9624.45	1.300	0.0013
MYC	129	9745	91	9783	1.423	0.0061
PAX5	194	9680	151.35	9722.65	1.287	0.0113
EGR1	85	9789	61.55	9812.45	1.384	0.0343
T	189	9685	155.95	9718.05	1.216	0.0411

We also downloaded binding motifs for 118 TFs from TRANSFAC database [14]. There are 30 genes with odds ratio scores above 1.20 and they will also be used for the GO term analysis in the next subsection. Table 3 shows 10 genes with the highest odds ratio scores.

**Table 3:** The numbers of FIMO hits of mouse TFs from TRANSFAC database with top 10 *O*_*hc*_, ranked by the p-values of the odds ratios (PRDM9's results are also shown in this table)

*HG *Genes	*HM*	*HN*	*CM*	*CN*	*O_hc_*	p-value
MYOD1	483	9391	341.05	9532.95	1.434	*<*0.0001
PRDM9	1405	8469	1120.35	8753.65	1.30	*<*0.0001
MYC/MAX	98	9776	58.25	9815.75	1.689	0.0009
USF2	111	9763	70.05	9803.95	1.591	0.0014
ATF4	79	9795	44.55	9829.45	1.780	0.0015
USF1	101	9773	63.45	9810.55	1.598	0.0019
AHR	97	9777	62.3	9811.7	1.563	0.0034
ARNT	91	9783	60.2	9813.8	1.516	0.0071
ETS1	64	9810	40.95	9833.05	1.567	0.0157
ATF4	67	9807	44.35	9829.65	1.514	0.0181
CNTN2	43	9831	27.6	9846.4	1.56	0.0480

### GO term analysis

Before analyzing the GO enrichment in *HG *genes, we first compute their semantic similarity to two manually-collected terms, namely "DNA recombination" (GO:0006310) and "Meiosis" (GO:0007127), which are highly related to recombination hotspots. Table 4 shows the semantic similarity between JASPAR *HG *genes and these two recombination related terms. PRDM9 has higher similarity score than all the genes in JASPAR, which confirms the recent discovery that it is a major trans-regulator of recombination hotspots [2-4]. Meanwhile, 13 genes in Table 4 have an average similarity score 0.192, which is higher than that of all the JASPAR genes together with PRDM9 (0.173). Thus, genes with higher odds ratio scores have higher semantic similarity to recombination-related terms, demonstrating that those odds ratio scores are indeed of help for selecting trans-regulator candidates.

**Table 4:** Semantic similarity between JASPAR *HG *genes and two recombination related terms

Genes	Similarity scores
PRDM9	0.327
SPZ1	0.319
CTCF	0.249
PAX5	0.182
GABPA	0.180
EGR1	0.175
ESRRB	0.166
MYC	0.166
KLF4	0.163
RXRA	0.157
ZFX	0.145
T	0.141
MYCN	0.126

Next, we apply the gap score in Equation 3 for GO term enrichment analysis. Our GO analysis shows that genes with high preference of binding to hotspots are enriched with epigenetic functions. As shown in Table 5, all the top 10 GO terms are directly related with epigenetic regulation (e.g. histone modification, DNA methylation, chromatin modification). More interestingly, the top 17*^th ^*term (GO:0007283, not shown in this table) is spermatogenesis, which suggests that predicted candidate genes are related with the generation of male gamete, confirming the key role of meiotic recombination hotspots in sexual reproduction. Another GO term of interest (GO: 0032204, not shown in Table 5) is ranked 19, namely regulation of telomere maintenance, which suggests a link with chromosome organization. Similarly, epigenetic terms are also enriched in trans-regulators predicted from TRANSFAC database as shown in Table 6. We also find that as the gap score becomes smaller down the list, the proportion of epigenetic terms becomes lower, and none of the 10 GO terms with the lowest gap scores is related with epigenetics. Therefore, the gap scores of epigenetic GO terms are associated with the odds ratios of candidate genes indicating preference of binding to hotspots. The functional connection with epigenetics observed here is consistent with the discovery of PRDM9, which is itself a histone methyltransferase. Indeed, much attention has been paid to epigenetic regulatory mechanisms of recombination hotspots (see the review [18] and references therein). Our approach and results in this paper would bring additional insights into the epigenetic control of recombination hotspots.

**Table 5:** GO terms enriched in JASPAR TFs with high odd ratio scores (with top 15 *gap *scores)

Rank	GO terms	GO term descriptions	*gap*
1	GO:0016571	histone methylation	0.282
2	GO:0018022	peptidyl-lysine methylation	0.203
3	GO:0051573	negative regulation of histone H3-K9 methylation	0.201
4	GO:0031060	regulation of histone methylation	0.184
5	GO:0051574	positive regulation of histone H3-K9 methylation	0.168
6	GO:0016568	chromatin modification	0.165
7	GO:0051571	positive regulation of histone H3-K4 methylation	0.165
8	GO:0006338	chromatin remodeling	0.157
9	GO:0035065	regulation of histone acetylation	0.148
10	GO:0006306	DNA methylation	0.146
11	GO:0010216	maintenance of DNA methylation	0.143
12	GO:0016584	nucleosome positioning	0.135
13	GO:0016485	protein processing	0.121
14	GO:0031065	positive regulation of histone deacetylation	0.119
15	GO:0018108	peptidyl-tyrosine phosphorylation	0.117

**Table 6:** GO terms enriched in TRANSFAC TFs with high odd ratio scores (with top 15 *gap *scores)

Rank	GO terms	GO term descriptions	*gap*
1	GO:0016571	histone methylation	0.105
2	GO:0051574	positive regulation of histone H3-K9 methylation	0.0927
3	GO:0031060	regulation of histone methylation	0.0925
4	GO:0051571	positive regulation of histone H3-K4 methylation	0.0916
5	GO:0051573	negative regulation of histone H3-K9 methylation	0.0865
6	GO:0000432	positive regulation of transcription from RNA polymerase II promoter by glucose	0.0839
7	GO:0043619	regulation of transcription from RNA polymerase II promoter in response to oxidative stress	0.0826
8	GO:0006357	regulation of transcription from RNA polymerase II promoter	0.0791
9	GO:0035065	regulation of histone acetylation	0.0786
10	GO:0031065	positive regulation of histone deacetylation	0.0766
11	GO:0018022	peptidyl-lysine methylation	0.0764
12	GO:0006355	regulation of transcription, DNA-dependent	0.0763
13	GO:0000122	negative regulation of transcription from RNA polymerase II promoter	0.0746
14	GO:0046016	positive regulation of transcription by glucose	0.0738
15	GO:0045944	positive regulation of transcription from RNA polymerase II promoter	0.0732

### Case studies

We next introduce in details three genes with high preference of binding to hotspots which are also annotated with some of these top-ranked GO terms. First, ZFX is a zinc finger X-chromosomal protein and it is annotated with the term GO:0007283 (spermatogenesis). It is reported that ZFX mutation results in small animal size and reduced germ cell number in male and female mice. Second, MYC with the term GO:0032204 (regulation of telomere maintenance) is a transcription factor that is believed to regulate expression of 15% of all genes through binding to Enhancer Box sequences (E-boxes) and recruiting histone acetyltransferases (HATs). In addition to its role as a classical transcription factor, MYC also functions to regulate global chromatin structure by regulating histone acetylation. Third, CTCF with both the terms GO:0010216 and GO:0006306 is a sequence-specific DNA-binding transcriptional regulator, insulator, and organizer of higher-order chromatin structure. It contains 11 *C*_2_*H*_2_-type zinc fingers. It is involved in promoter activation or repression, hormone-responsive gene silencing, methylation-dependent chromatin insulation, and genomic imprinting and mediates pairing between × chromosomes and interactions between distant regulatory elements. Interestingly, the KFL4 gene, which has top odds ratio score and p-value in Table 2, does not show epigenetic functions. It is annotated with two GO terms, namely GO:0006355 (regulation of transcription, DNA-dependent) and GO:0045892 (negative regulation of transcription, DNA-dependent) which also have high gap scores. It might imply that recombination hotspots are regulated in concert by both epigenetic and DNA-dependent mechanisms.

### Analysis of hotspot coverage

In this subsection, we aim to analyze how well those top-ranked genes cover current known hotspots. Given a gene *g*, *HS*(*g*) is the set of hotspots covered by *g*. As shown in Table 1, PRDM9 covers 1405 hotspots, i.e. its *HM *number = |*HS*(PRDM9)| = 1405. Table 7 shows the number of hotspots covered by JASPAR *HG *genes and the number of common hotspots covered by PRDM9 and those *HG *genes. For example, the gene T covers 189 distinct hotspots and 24 of them are also covered by PRDM9.

**Table 7:** Hotspot coverage of JASPAR TFs with high odd ratio scores (JASPAR *HG *genes). The second column (*HM*) shows the number of hotspots covered by the JASPAR *HG *gene in the first column. The third column (| ∩ *HS*(PRDM9) |) shows the number of common hotspots covered by PRDM9 and the JASPAR *HG *gene

Genes in *HG*	*HM*	| ∩ *HS*(PRDM9) |
T	189	24
PAX5	194	47
KLF4	886	177
GABPA	110	23
RXRA	1015	157
MYCN	197	44
SPZ1	322	63
CTCF	1002	163
ESRRB	792	110
ZFX	437	112
MYC	129	29
EGR1	85	21

For further analysis, we built a hotspot coverage graph *HC *= (*V*, *E*, *w*) where nodes are PRDM9 and all the JASPAR *HG *genes and edges show the hotspot coverage similarity between nodes. In particular, *V *= {*PRDM*9} ∪ *HG *and each pair of nodes has a weight, indicating their hotspot coverage similarity, based on the meet/min coefficient [19] in equation 4.

(4) $$w\left(g_{i},g_{j}\right)=\frac{\left|HS\left(g_{i}\right)\cap HS\left(g_{j}\right)\right|}{\text{min}\left\{\left|HS\left(g_{i}\right)|,|HS\left(g_{j}\right)\right|\right\}},\forall g_{i},g_{j}\in V.$$

**Figure 4:**
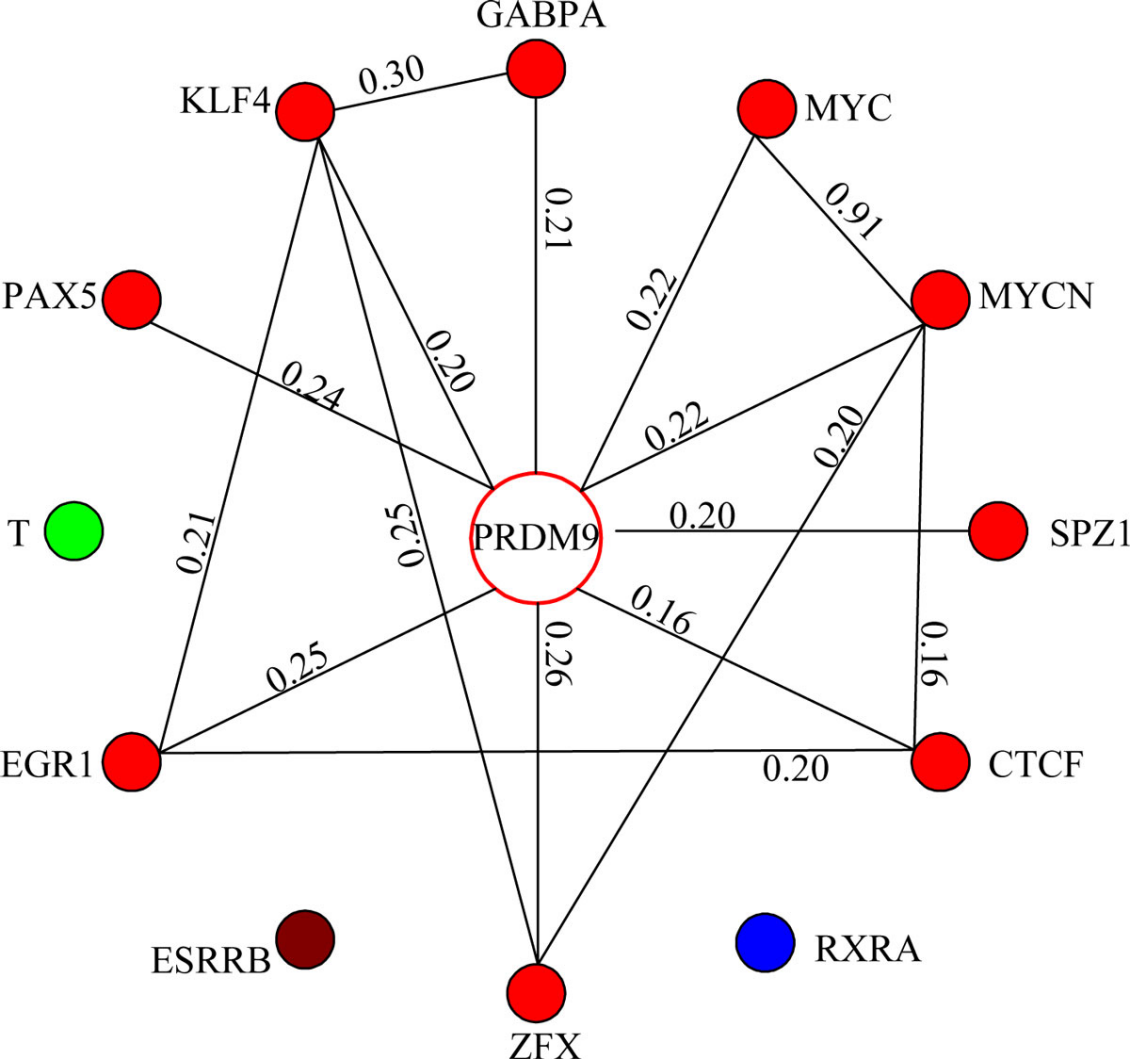
**Hotspot coverage graph**. Figure 4 shows the hotspot coverage graph where each node is a gene with high odds ratio score and edges between nodes represent their hotspot coverage similarities. Here each color stands for a gene cluster.

In this hotspot coverage graph, we divide genes into several clusters and genes in the same cluster will have higher hotspot coverage similarities. A simple solution for clustering [20] is as follows. We first set a threshold *w_t _*and filter all the edges with weights lower than *w_t_*. The remaining connected components are considered as gene clusters. In our experiments, we gradually increased the threshold *w_t _*and obtained a hotspot coverage graph with 4 clusters as shown in Figure 4 when *w_t _*= 0.16. Another solution is to apply hierarchical clustering algorithm. When we set the number of clusters we want to obtain as 4, the 4 clusters of hierarchical clustering are exactly the same as those in Figure 4. In the cluster with 10 red TFs including PRDM9 in Figure 4, all the other TFs have edges to PRDM9 with weights larger than or equal to 0.16, indicating that they all share similar hotspot coverage patterns with PRDM9. Meanwhile, three singleton clusters consist of TFs with the lowest odds ratios as shown in Table 2 and they have no edges to PRDM9. These two observations confirm once again that PRDM9 is a major trans-regulator for mouse hotspots.

**Figure 5:**
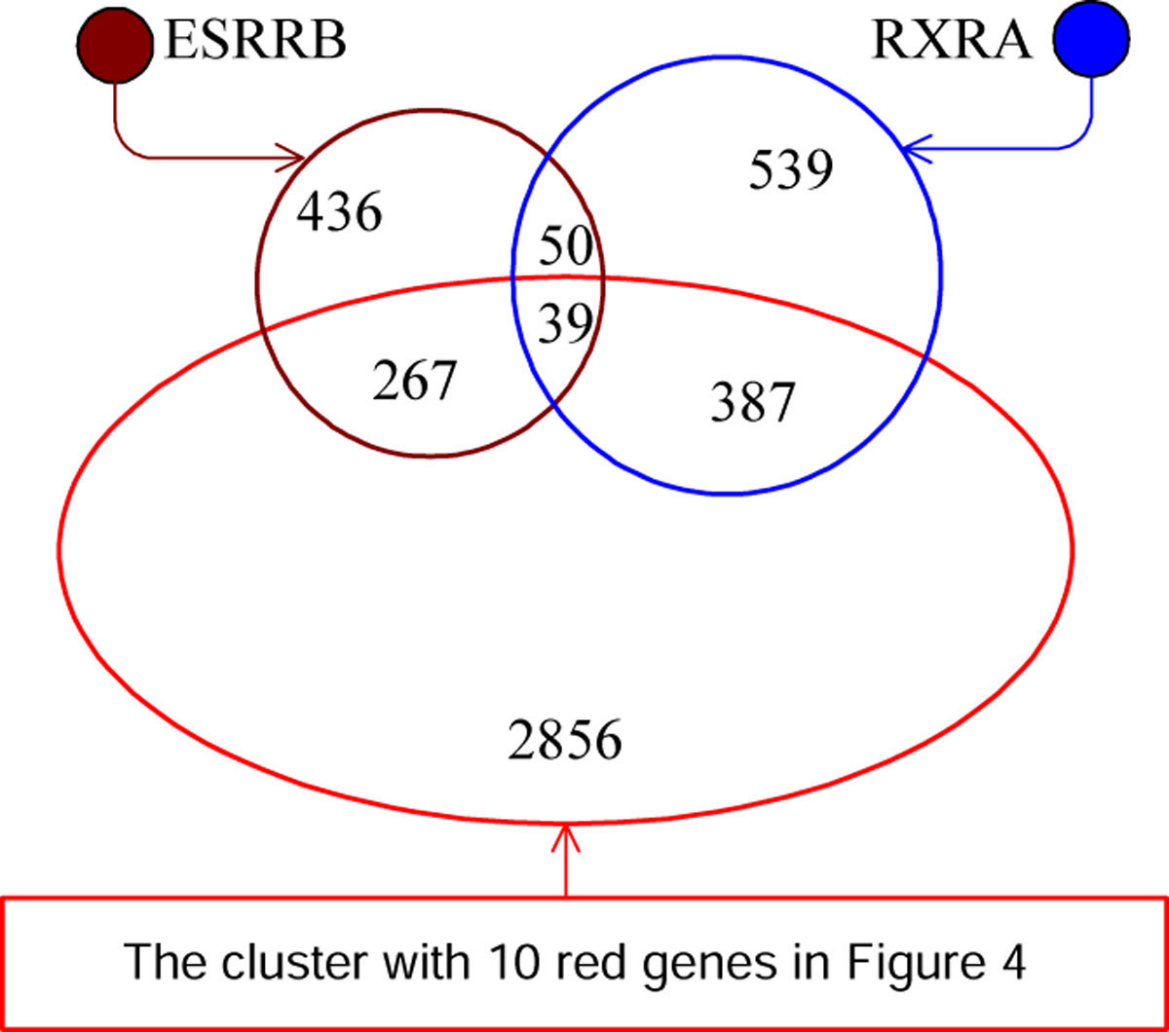
**Venn diagram of the hotspot coverage**. Figure 5 shows the hotspot coverage of the above 4 clusters in Figure 4.

We also show the hotspot coverage of the above 4 clusters in Figure 5. Compared with other clusters, the cluster with only the gene T covers a much smaller number of hotspots and it is thus not shown in Figure 5. In this figure, the red cluster with 10 TFs covers 3549 hotspots and shares common 39 hotspots with other two clusters. In addition, the number of hotspots covered by at least one of the 4 clusters in Figure 4 is 4679. The fact that many hotspots (i.e., 5195 out of 9874 known hotspots) are still not covered by PRDM9 or motifs in our study suggests that we need to search for additional proteins and motifs in the future for a higher coverage for hotspots.

## Conclusions

In this paper, we proposed a new approach to discovering trans-regulators of recombination hotspots in mouse genome. Starting from experimentally identified or predicted binding sites of DNA binding proteins, we scan the DNA sequences of hotspots and coldspots for target binding occurrences of each protein. The relative enrichment of binding targets in hotspots is used to estimate the likelihood that a protein has regulatory effect on recombination hotspots. We increased the rigor by designing a GO analysis method to identify shared functions of candidate genes. Applying our method to newly mapped genome-wide mouse recombination hotspots, we confirmed the recent discovery that PRDM9 is a major trans-regulator of recombination hotspots. Further, we identified a list of additional proteins as candidate trans-regulators. GO analysis shows that the most prominent functions shared by these candidate genes are histone modifications, which confirms and provide new insights into the epigenetic mechanism of recombination hotspots. Thus the approach developed in this paper can be used to identify additional trans-regualtors of hotspots. The predicted proteins and their functional analysis can shed light on the pathways (rather than the single gene of PRDM9) regulating recombination, and be used to guide further experimental studies of recombination hotspots.

Currently, the number of proteins examined in this paper is small (i.e. only 53 transcription factors in mouse genome from JASPAR database and 118 from TRANSFAC database). In the future, we will try to collect more DNA-binding proteins from more sources for more comprehensive results. Meanwhile, we searched for target binding sites of proteins using FIMO, which does not allow for insertion and deletions in motif matching. However, it is known that the DNA target sites of some proteins contain indels [21]. Therefore, more flexible motif finding algorithms that take into account special sequence patterns (e.g. nucleotide adjacent dependency [22]) may be used to address this problem. Although the recombination hotspots analyzed in this paper was obtained experimentally, our approach is not limited to this type of data and we can computationally estimate recombination rates from sequence polymorphism data in large scale, either based on LD structure [23,24] or pedigree structure [25]. In addition, as our results of GO analysis suggested that epigenetic mechanism is shared by top candidate genes, we will follow up with studies of epigenetic interaction between histone and DNA as mediated by PRDM9 and other predicted proteins.

## Competing interests

The authors declare that they have no competing interests.

## Authors' contributions

MW and JZ conceptualized and designed the method and drafted the manuscript together. MW was responsible for the implementation. JL, CKK and TMP participated in discussion as well as revising the draft. All authors read and approved the manuscript.
